# A novel diagnostic technique in para-Hisian pacing under rapid adenosine triphosphate injection: A rare case of septal accessory pathway in a coronary sinus diverticulum

**DOI:** 10.1016/j.hrcr.2022.08.012

**Published:** 2022-09-02

**Authors:** Koji Sudo, Tetsuya Asakawa, Moeko Abe, Kazuya Nakagawa, Kenji Kuroki, Akira Sato

**Affiliations:** ∗ Department of Cardiovascular Medicine, University of Yamanashi, Yamanashi, Japan; † Department of Cardiology, Yamanashi Kosei Hospital, Yamanashi, Japan

**Keywords:** Adenosine triphosphate, Coronary sinus diverticulum, Para-Hisian pacing, Septal accessory pathway, Supraventricular tachycardia

**Key Teaching Points**

• To take care in performing para-Hisian pacing (PHP) when there are 2 retrograde conductions over an atrioventricular node and an accessory pathway (AP).
• PHP under rapid intravenous adenosine triphosphate injection is a useful method for clarifying retrograde conduction of the AP.
• Coronary sinus (CS) diverticulum is a major CS anomaly. It is important to evaluate CS anatomy using contrast-enhanced imaging before catheter ablation of supraventricular arrhythmias.

## Introduction

Adenosine triphosphate (ATP) is used in electrophysiological studies (EPS). Its uses include the diagnosis and treatment of supraventricular tachycardia (SVT), diagnosis of ATP-sensitive atrial tachycardia, and confirmation of dormant pulmonary vein conduction after pulmonary vein isolation during the acute phase.

Para-Hisian pacing (PHP) is a useful method for distinguishing retrograde conduction over an atrioventricular (AV) node (AVN) or an accessory pathway (AP) for maneuvering during EPS. Although it is a simple technique, there are some limitations in interpreting the potentials. We report a novel technique in PHP under rapid intravenous ATP injection and successful radiofrequency (RF) ablation of a septal AP in the coronary sinus (CS) diverticulum (CSD).

## Case report

A 30-year-old man referred to our hospital provided written informed consent for EPS and catheter ablation. The patient had experienced frequent palpitation episodes with a regular narrow QRS complex tachycardia and heart rate of 220 bpm, which was terminated by administration of 20 mg of ATP. Baseline electrocardiogram showed sinus rhythm with intermittent ventricular pre-excitation. Ventricular pre-excitation was positive in lead I, flat in lead II, and negative in leads III, aVF and V_1_, which we assumed was localized in the septal AP. The 24-hour Holter recording showed sudden-onset SVT with palpitations and revealed short RP tachycardia triggered by an atrial premature contraction. The patient had no significant comorbidities. Echocardiography showed normal heart structure and no Ebstein anomaly or persistent left superior vena cava.

**Figure 1:**
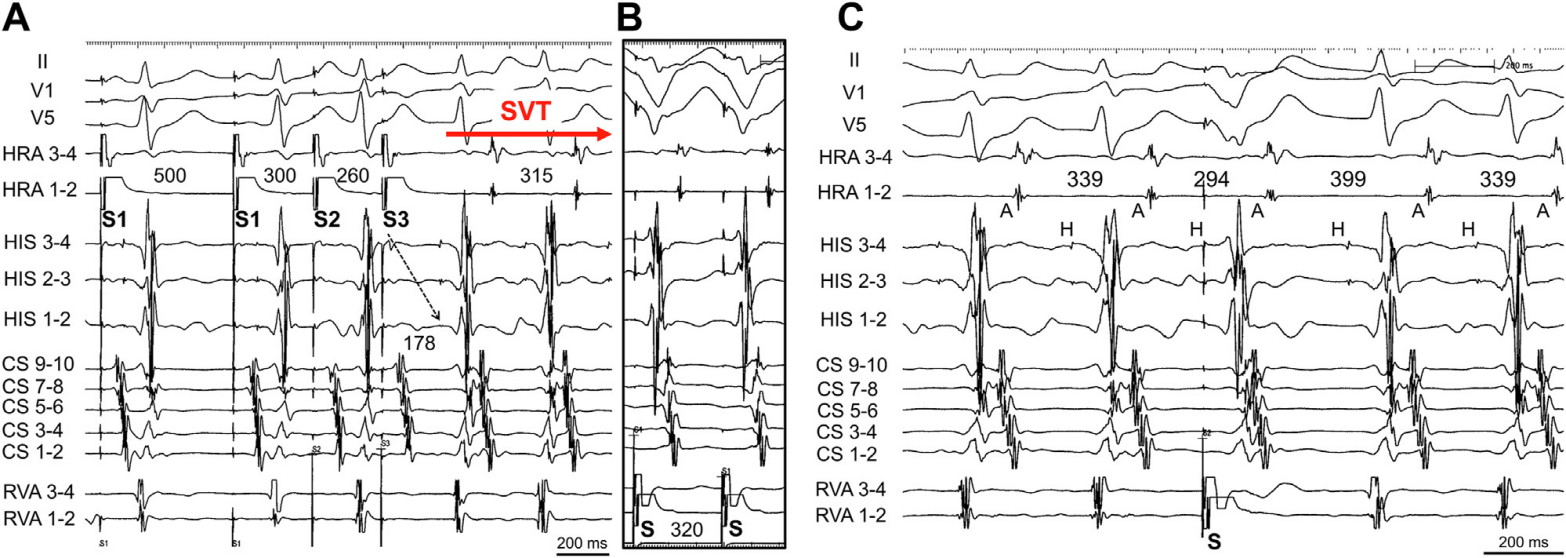
Findings of electrophysiological study. **A:** Supraventricular tachycardia (SVT) induced by atrial premature stimulation (S1-S1 500 ms; S1-S2 300 ms; S2-S3 260 ms). **B:** Ventricular pacing showed the same retrograde atrial sequence. **C:** Premature ventricular stimulus during the His refractory period reset the SVT. CS = coronary sinus; HRA = high right atrium; RVA = right ventricular apex; S = stimulation.

**Figure 2:**
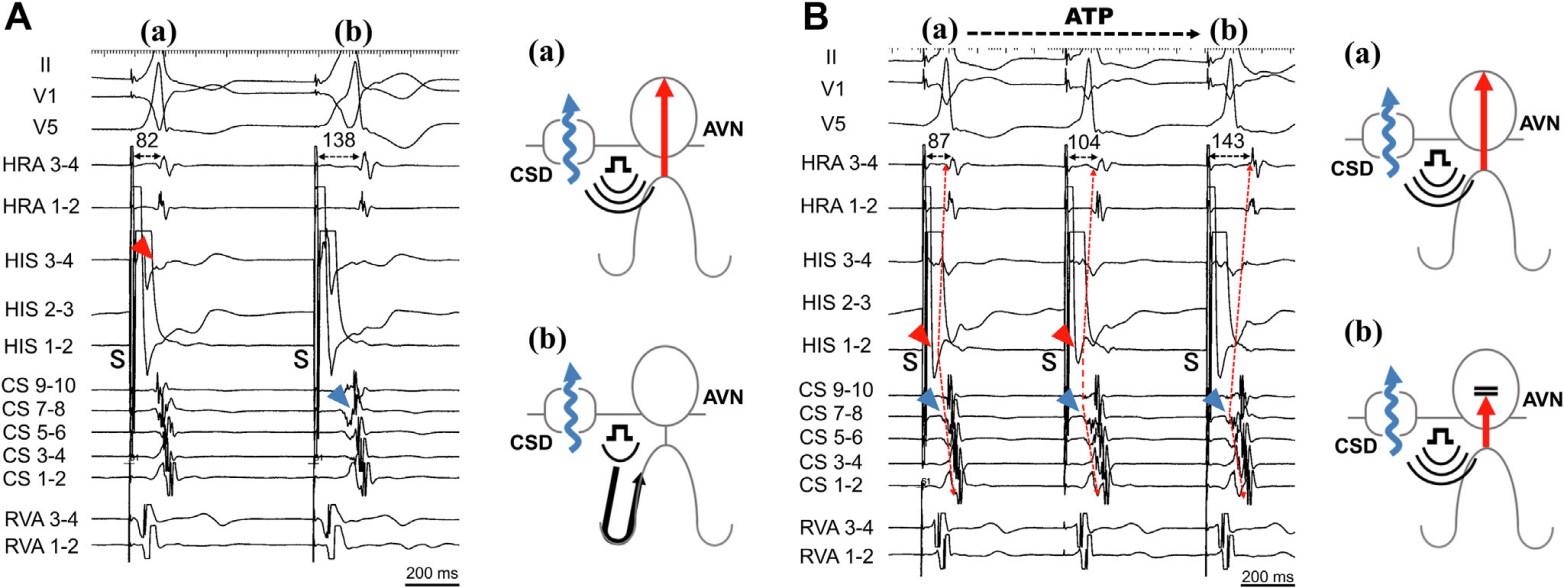
Para-Hisian pacing (PHP) before catheter ablation and diagrams. **A:** PHP showed an atrioventricular nodal (AVN) pattern, but the atrial sequence is different for narrow and wide QRS. The earliest atrial sequence changed from His *(red arrowhead)* to CS *(blue arrowhead).* Both **(a)** and **(b)** show 2 pattern diagrams of PHP. **B:** PHP under rapid intravenous adenosine triphosphate (ATP) injection. The earliest atrial sequence changed from His *(red arrowheads)* to CS *(blue arrowheads)* with the atrial sequence change *(red dotted arrows),* but both show narrow QRS morphology. Both **(a)** and **(b)** show 2 pattern diagrams of PHP, respectively. CSD = coronary sinus diverticulum; other abbreviations as in Figure 1.

**Figure 3:**
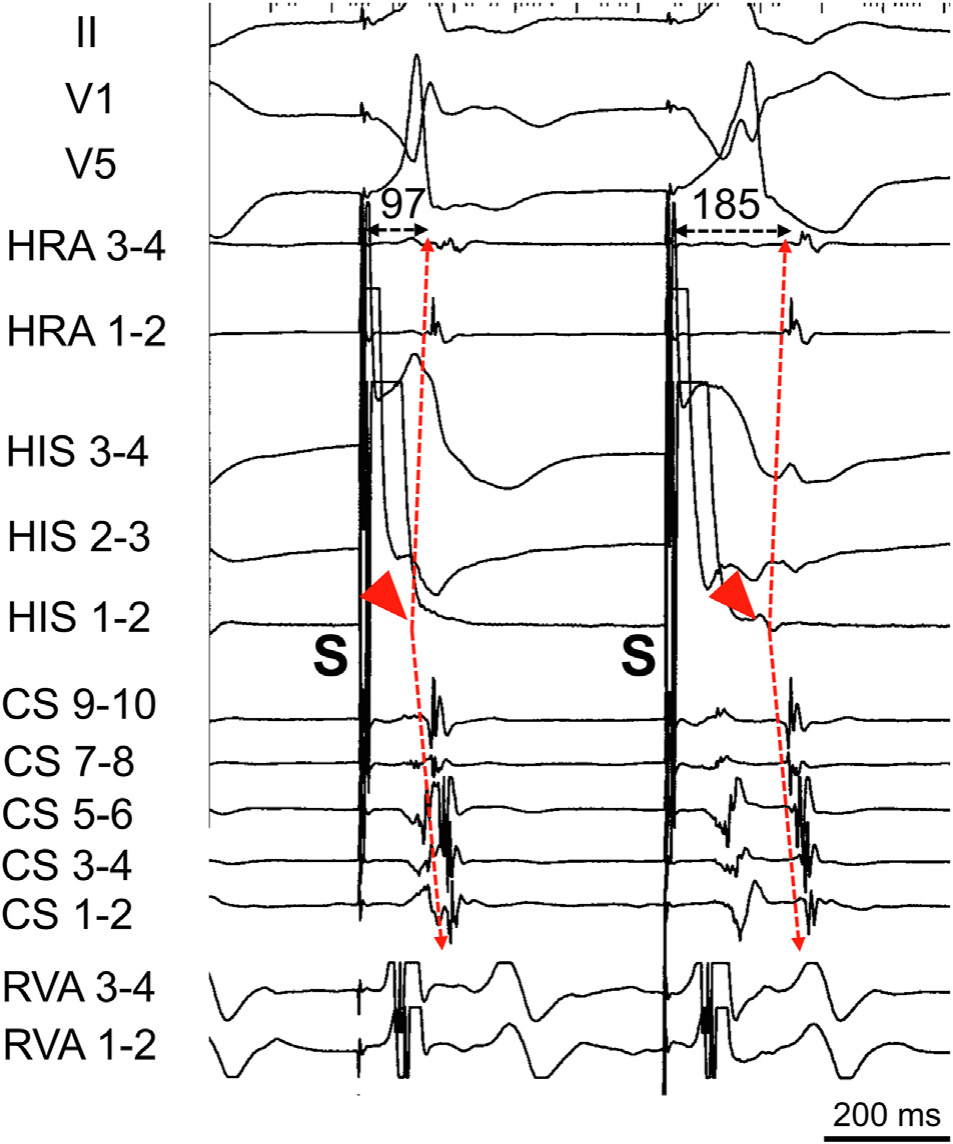
Para-Hisian pacing after catheter ablation. The stimulus-atrial interval was prolonged to 88 ms, with the same atrial sequence *(red dotted arrows)* from the narrow to the wide QRS during parahisian pacing after radiofrequency ablation. The earliest atrial sequence was still His *(red arrowheads).* An atrioventricular nodal pattern is shown. Abbreviations as in Figure 1.

Four electrode catheters were positioned in the high right atrium, right ventricular apex, His-bundle region, and CS. AH and HV intervals were normal at baseline. During atrial premature stimulation (S1-S1 500 ms; S1-S2 300 ms) from the high right atrium, when S2-S3 was shortened from 270 to 260 ms, SVT (tachycardia cycle length 315 ms) was induced (Figure 1A) and continued without ventriculoatrial (VA) block. The atrial electrogram sequence during SVT was the same as that during ventricular pacing (Figure 1B). Burst pacing from the right ventricular apex showed entrainment of the atrium and a V-A-V response, which was unlikely to be atrial tachycardia. A premature ventricular stimulus delivered during the His refractory period reset the following His-bundle activation during SVT (Figure 1C), which was unlikely to have been caused by atrioventricular nodal reentrant tachycardia (AVNRT). When PHP was performed with high output followed by gradual reduction, QRS wave morphology changed from narrow to wide. At first glance, it appeared to be an AVN pattern, but the atrial sequence showed that, during PHP, the earliest site of atrial sequence changed from the His recording site to CS7-8 (Figure 2A). Therefore, to distinguish the retrograde conduction pathway, we performed PHP under rapid intravenous ATP injection with a dose of 10 mg. The earliest atrial sequence changed from His to CS, but both had narrow QRS morphology (Figure 2B), a finding that proved the existence of retrograde AP with conduction block of retrograde AVN conduction. Based on the EPS findings, we diagnosed the SVT as an orthodromic reentrant tachycardia. We used a 3.5-mm, irrigated-tip contact force–sensing ablation catheter (TactiCath, Sensor Enabled, Abbott, Abbott Park, IL) for RF ablation. CS angiography was performed via a steerable sheath (Agilis Medium Curl, Abbott). A CSD was observed after injection of contrast medium at the location of the successful ablation site. The electrogram of the ablation catheter at the CSD was 31 ms earlier than the earliest atrial electrogram. Before RF application, coronary angiography confirmed the coronary arteries so as to avoid conductive heating. RF ablation, limited to 30 W with contact force maintained between 10*g* and 15*g*, was performed during SVT. At this site, RF application was delivered. Six seconds after starting the first RF application with a power of 20 W, VA block occurred and the tachycardia was terminated. The RF application was delivered for a total of 60 seconds. After catheter ablation, programmed stimulation was performed with isoproterenol infusion. However, SVT was never induced, and the septal AP disappeared during bolus intravenous ATP injection with a dose of 20 mg. The stimulus-atrial interval was prolonged with the same atrial sequence from the narrow QRS to the wide QRS during PHP, which showed an AVN conduction pattern (Figure 3). The AH and HV intervals were normal after catheter ablation, and no other complications occurred. Three months after catheter ablation, a 24-hour Holter recording showed normal sinus rhythm without ventricular pre-excitation. The patient has experienced no tachyarrhythmias for 5 years after catheter ablation.

## Discussion

PHP, first reported by Hirao et al^1^ in 1996, is a useful pacing method for distinguishing retrograde AP conduction from retrograde AVN conduction in EPS; however, this simplified algorithm has some limitations in explaining EPS phenomena. For example, atypical capture patterns such as inadvertent atrial capture and pure His-bundle capture need to be considered.^2^^,^^3^ Moreover, if there are 2 retrograde conductions via the AVN and AP, then the following situations need to be considered to explain complex PHP phenomenon: (1) is the AP at a distance from the pacing site (eg, left free wall Kent); and (2) even if the AP is in the septum, is this a case of AP conduction velocity being slow, such as AP with slower retrograde conduction than AVN and slow Kent. Hirao et al^4^ reported on the importance of morphological change on the atrial electrogram during PHP. This case had 2 retrograde conductions via the AVN and septal AP. At first glance, PHP showed an AVN pattern; however, there was a slight difference in the atrial electrogram sequence during PHP. Rapid intravenous ATP injection led to the accurate diagnosis of SVT due to transient retrograde conduction block of the AVN.

Differences in conduction characteristics between the AVN and AP usually are useful for differentiating the retrograde pathway. However, in this case, even if pacing output and pacing rate were adjusted, neither the AVN nor the septal AP could have been selectively and stably conducted, and discrimination by this method was not possible. Therefore, PHP under rapid intravenous ATP injection was performed by utilizing retrograde conduction block of the AVN. To the best of our knowledge, this is the first case of PHP under rapid intravenous ATP injection to prove retrograde septal AP.

### Study limitations

Decremental conducting APs, such as the Mahaim pathway, have been reported. The Mahaim fiber was first described by Mahaim and Benatt^5^ in 1938 as a tissue leading from the AVN to the ventricles. Ellenbogen et al^6^ demonstrated the response to conduction over the Mahaim pathway by administering various drugs and showed that adenosine could induce conduction block over the Mahaim pathway. Okishige et al^7^ also reported the response of the AV and atriofascicular pathways to ATP. After intravenous administration of 10 mg of ATP, complete conduction block of the Mahaim pathway, as well as of the AVN, occurred. Moreover, slow Kent has been reported, especially as an AP with slow conduction and decremental properties. Histologic studies of slow Kent fascicles suggest they contain AVN-like tissue, so they are thought to exhibit a decremental property.^8^ Therefore, slow Kent also is ATP-sensitive. In these cases in which an AP exhibits special conduction characteristics, it may be difficult to distinguish between distinct kinds by selective disruption of only AVN conduction by PHP under rapid intravenous ATP injection. The AP in this case did not exhibit decremental property conduction, and VA conduction did not occur as slowly as in slow Kent.

This was also a rare case because the septal AP was successfully ablated in the CSD. The 3 major CS anomalies are (1) CSD; (2) persistent left superior vena cava; and (3) enlarged CS ostium. Diverticula with posteroseptal APs were first reported in 1985^9^ and subsequently have been reported many times. Diverticulum of the proximal CS has been reported in 7%–11% of patients with posteroseptal APs.^10^ The diverticulum has various morphologies,^11^ and this case showed a medium, wide-necked diverticulum on CS angiography. Thus, it is important to perform contrast-enhanced evaluation of the CS anatomy before EPS and catheter ablation of SVT.

## Conclusion

In cases of septal AP, a preliminary understanding of the CS anatomy is important for increasing the success of catheter ablation. PHP is an EPS maneuver necessary for determining the mechanism of SVT. If there are 2 retrograde conductions over the AVN and AP, a novel diagnostic technique in PHP under rapid intravenous ATP injection can clarify the retrograde conduction of AP.

## References

[1] Hirao K., Otomo K., Wang X. (1996). Para-Hisian pacing: a new method for differentiating retrograde conduction over an accessory AV pathway from conduction over the AV node. Circulation.

[2] Takatsuki S., Mitamura H., Tanimoto K. (2006). Clinical implication of “pure” Hisian pacing in addition to para-Hisian pacing for the diagnosis of supraventricular tachycardia. Heart Rhythm.

[3] Ali H., Foresti S., Lupo P. (2019). Para-Hisian pacing: new insights of an old pacing maneuver. JACC Clin Electrophysiol.

[4] Hirao K., Yamamoto N., Toshida N. (2000). Diagnostic significance of the morphological change in the atrial electrogram during para-Hisian pacing. Jpn Circ J.

[5] Mahaim I., Benatt A. (1938). Nouvelles recherches sur les connexions supérieures de la branche gauche du faisceau de His-Tawara avec cloison interventriculaire. Cardiologia.

[6] Ellenbogen K.A., Rogers R., Old W. (1989). Pharmacological characterization of conduction over a Mahaim fiber: evidence for adenosine sensitive conduction. Pacing Clin Electrophysiol.

[7] Okishige K., Goseki Y., Itoh A. (1998). New electrophysiologic features and catheter ablation of atrioventricular and atriofascicular accessary pathways: evidence of decremental conduction and the anatomic structure of the Mahaim pathway. J Cardiovasc Electrophysiol.

[8] Klein G.J., Prystowsky E.N., Pritchett E.L., Davis D., Gallagher J.J. (1979). Atypical pattern of retrograde conduction over accessory atrioventricular pathways in the Wolff-Parkinson-White syndrome. Circulation.

[9] Gerlis L., Davies M., Boyle R., Williams G., Scott H. (1985). Pre-excitation due to accessory sinoventricular connexions associated with coronary sinus aneurysms. A report of two cases. BMJ Heart.

[10] Payami B., Shafiee A., Shahrzad M., Kazemisaeed A., Davoodi G., Yaminisharif A. (2013). Posteroseptal accessory pathway in association with coronary sinus diverticulum: electrocardiographic description and result of catheter ablation. J Interv Card Electrophysiol.

[11] Sun Y.X., Arruda M., Otomo K. (2002). Coronary sinus-ventricular accessory connections producing posteroseptal and left posterior accessory pathways: incidence and electrophysiological identification. Circulation.

